# Visual Detection of Stem-Loop Primer Amplification (SPA) Products without Denaturation Using Peroxidase-like DNA Machines (PxDM)

**DOI:** 10.3390/ijms24097812

**Published:** 2023-04-25

**Authors:** Yulia I. Maltzeva, Daria A. Gorbenko, Ekaterina V. Nikitina, Maria S. Rubel, Dmitry M. Kolpashchikov

**Affiliations:** 1 Laboratory of Solution Chemistry of Advanced Materials and Technologies, ITMO University, 191002 St. Petersburg, Russia; maltseva.yulia8@gmail.com (Y.I.M.); gorbenko@scamt-itmo.ru (D.A.G.); 2 Pediatric Research and Clinical Center for Infectious Diseases, 197022 St Petersburg, Russia; 3 Chemistry Department, University of Central Florida, 4000 Central Florida Boulevard, Orlando, FL 32816, USA; dmitry.kolpashchikov@ucf.edu; 4 Burnett School of Biomedical Sciences, University of Central Florida, Orlando, FL 32816, USA; 5 National Center for Forensic Science, University of Central Florida, Orlando, FL 32826, USA

**Keywords:** point-of-care diagnostics, detection of dsDNA amplicons, G-quadruplex, isothermal amplification, DNA-nanomachine, single nucleotide selectivity

## Abstract

Rapid, inexpensive, and accurate determination of nucleic acids is a decisive factor in evaluating population’s health and monitoring treatment at point-of-care (POC) settings. Testing systems with visual outputs can provide instrument-free signal detection. Isothermal amplification technologies can substitute conventional polymerase chain reaction (PCR) testing due to compatibility with the POC diagnostic. Here, we have visually detected DNA fragments obtained by stem-loop-primer-assisted isothermal amplification (SPA), but not those obtained by PCR or LAMP amplification using DNA nanomachines with peroxidase-like activity (PxDM) with sensitivity to a single nucleotide substitution. Compared to the diagnostics with conventional loop-mediated isothermal amplification (LAMP), the PxDM method produces no false positive signals with the non-specific amplification products. The study suggests that PxDM, in conjunction with SPA isothermal amplification, can become a valid platform for POC testing systems.

## 1. Introduction

Prompt identification of causative agents for infectious disease at early stages allows implementing appropriate and timely treatment to avoid the development of complications [1]. The diagnostics based on sequencing and quantitative PCR is widely used and is the gold standards in clinical research due to their versatility, high reliability, and unique sensitivity. Unfortunately, this technology has such limitations as high cost of reagents, complex and expensive equipment, specialized laboratory, and qualified personnel [2].

Isothermal amplification technologies allow nucleic acids to be amplified at a fixed temperature, opening new possibilities for developing portable, low-cost point-of-care (POCT) devices for molecular diagnostics with high sensitivity. There have appeared several POCT-compatible amplification techniques, including LAMP [3,4], nucleic acid sequence-based amplification [5], rolling cycle amplification [6] and strand displacement amplification [7]. LAMP as one of the most sensitive and robust amplification reactions is often accompanied by the accumulation of nonspecific amplification products [8,9] or false positive results caused by sensing small amounts of contamination [10]. Various methods have been proposed to improve the specificity of the reaction through additional fluorescent probes [11,12], chain displacement probes [13], touchdown protocol [14], chemical additives and improvements in primer design [10,14,15]. For instance, a new type of amplification called stem-loop-primer-assisted isothermal amplification (SPA) has been developed; due to a different primer design, it offers a reduced risk of false positives in comparison with LAMP [14].

Visual detection reactions that require no fluorescence readout devices can be used in developing test systems along with isothermal amplification to increase accessibility. Many detection technologies based on aptamers are used to detect nucleic acids [16]. Thus, sensors based on the G-quadruplexes (G-4) are widely used since the G-4/hemin complex can be visualized. This complex has peroxidase activity and can catalyze the oxidation of 3-3′-diaminobenzidine tetrahydrochloride (DAB) by H_2_O_2_ to produce a colorimetric signal detected by the naked eye [17]. The visualization strategy based on the DNAzyme was used to directly detect the DNA of viral [18] or bacterial [19,20] pathogens. More importantly, the split G-quadruplex DNAzyme strategy [21] can distinguish single-base differences, and has been used to differentiate gene mutations [19,21]. Equipping such a split construction with additional nucleic acid binding arms can enable the detection of folded RNA [22,23] or double-stranded DNA (dsDNA) [5] with high affinity and excellent selectivity towards signal base mismatch.

This article presents a combination of SPA and G-quadruplex-based PxDM to develop a highly accurate and selective diagnostic method after the isothermal SPA amplification of their genetic material. To the best of our knowledge, we have first achieved a visual (color change) detection of a dsDNA amplicon produced by SPA with *E. coli* as the model organism.

## 2. Results

### 2.1. Design of Peroxidase-like DNA Machines (PxDM)

**Figure 1:**
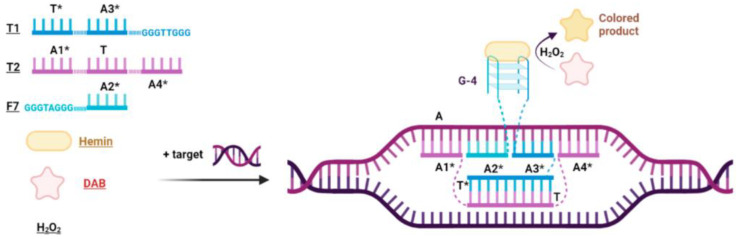
PxD DNA in complex with dsDNA analyte. Arms A1*, A3*, and A4*are responsible for unwinding. Arm A2* is responsible for high selectivity of DNA analyte recognition.

The design of PxDM is shown in Figure 1. The system consists of three oligonucleotides: T1, T2, and F7 (Table S2). Domains, or arms, A1*, A2*, A3*, and A4* of the T1, T2 and F7, have been designed complementarily, according to target sequence A. The T2 sequence is required for unwinding double-stranded DNA by hybridizing the A1* and A4* PxDM fragments to the target. This allows the A2* and A3* parts of the T1 and F7 sequences to selectively recognize the gene region. The structure of the DNA machine is fully assembled only when both G-rich sequences in T1 and F7 are close to each other and form the G-4 structure. The G-4 structure can bind hemin and form DNAzyme to catalyze the oxidation of a colorless substrate and generate a visual signal [5,17]. T and T* fragments associate into a dsDNA structure that stabilizes the PxDM in the solution. The F7 arm remains unbound to ensure the core formation only in a presence of analyte.

**Figure 2:**
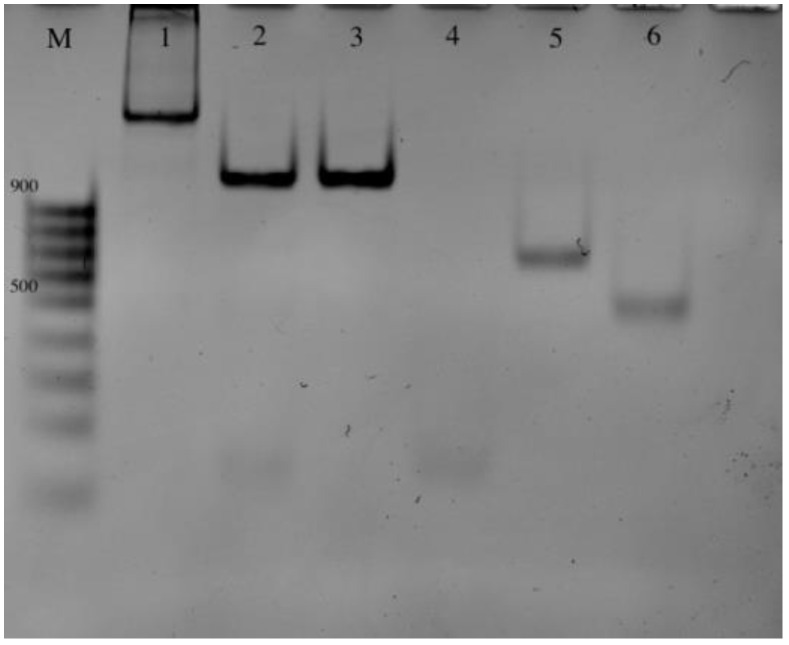
An analysis of the *E. coli* PxDM machine assembly in polyacrylamide gel electrophoresis: M—marker 100 bp+; 1—Assembled PxDM with the synthetic DNA; 2—assembled PxDM; 3—*E. coli* PxDM T1 and *E. coli* PxDM T2; 4—*E. coli* PxDM f7; 5—*E. coli* PxDM T2; 6—*E. coli* PxDM T1.

The correct assembly of the machine is confirmed via gel electrophoresis (Figure 2).

### 2.2. Amplification and Detection of the Target Fragments

**Figure 3:**
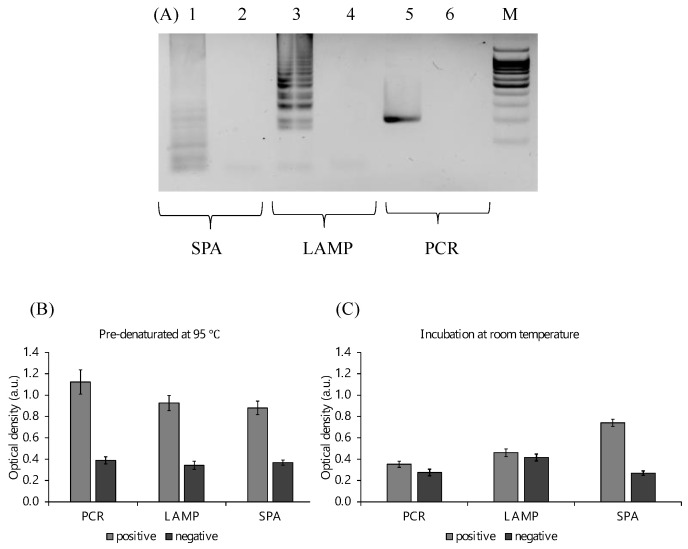
Visual detection of amplicons obtained by different amplification methods. (**A**) Analysis of the amplification products in 2% agarose gel: lane 1—SPA positive; lane 2—SPA negative (amplified SPA mixture with reagents and primers without bacterial RNA); lane 3—LAMP positive; lane 4—LAMP negative (amplified LAMP mixture with reagents and primers without bacterial RNA); lane 5—PCR positive; lane 6—PCR negative (amplified PCR mixture with reagents and primers without bacterial RNA); lane M—DNA size marker 100 bp+. (**B**) Colorimetric analysis of amplicon (300 ng each) detection with annealed at 95 °C followed by detection at room temperature. (**C**) Colorimetric analysis of amplicon (300 ng each) detection with incubation at room temperature without pre-denaturation.

PCR, LAMP, and SPA were selected to compare the detection efficiency of PxDM using different types of amplification (Figure 3A). In addition, the effect of denaturation before carrying out the reaction was investigated by the incubation of the solution at 95 °C. We found that PCR and LAMP amplification products can be detected only after the pre-denaturation of amplicons to the DNA machines (Figure 3B). At the same time, SPA amplification products were detected at room temperature without denaturation (Figure 3C), which is a more convenient POCT amplification method. In this regard, the SPA type of amplification was chosen for further developing the technology.

### 2.3. Optimization of the Peroxidase Reaction

**Figure 4:**
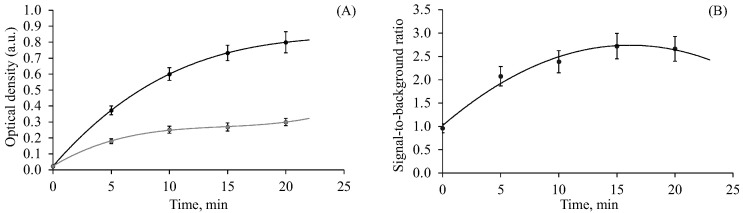
Optimizing the incubation time of the peroxidase reaction. (**A**) The effect of incubation time in the presence (target) or in the absence (no target) of the target DNA (**B**) The effect of incubation time on the signal-to-background ratio.

To determine the most suitable time to carry out the peroxidase reaction, the optical density of the solution was measured after different incubation time periods (Figure 4A). In addition, the signal-to-background ratio (S/B) was analyzed (Figure 4B). Thus, during the reaction, not only did the signal level increase but so did the background level leading to a decrease in the S/B. Thus, the most optimal incubation time with the greatest S/B was 15–20 min (Figure 4B). However, even after 5 min, we could clearly distinguish the signal from the background with the naked eye.

### 2.4. Limit of Detection (LOD) of the SPA/PxDM Assay

**Figure 5:**
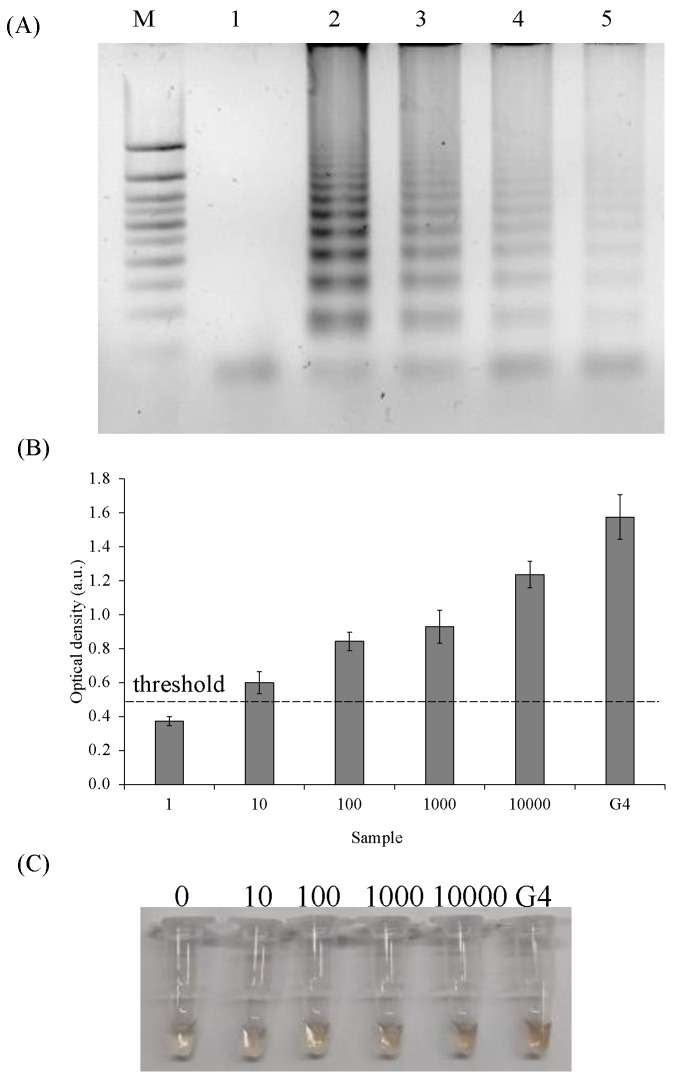
Sensitivity of PxD machine/SPA system. (**A**) SPA products analysis with various numbers of genomic equivalents (GE), 2% agarose gel: M—marker 50 bp+; 1—negative control; 2—10,000 GE; 3—1000 GE; 4—100 GE; 5—10 GE; (**B**) Colorimetric analysis of SPA product detection with various numbers of genomic equivalents. (**C**) The corresponding photographs of the colorimetric responses.

To assess the sensitivity of the SPA and the detection reaction, a serial dilution of *E. coli* DNA from 10,000 to 10 genomic equivalents (GE) was performed; the DNA samples were SPA amplified. The amplification products were analyzed in 2% agarose gel (Figure 5A). The amplification of various titers of *E. coli* genomic equivalents followed by the detection of 1 μL of the amplification mixture showed the possibility of identifying 10, 100, and 100 bacteria cells with S/B 2.3, 2.5, and 2.6, respectively, Figure 5B).

### 2.5. Selectivity of the SPA/PxDM Assay towards Different SPA Products and Single Nucleotide Mismatch

**Figure 6:**
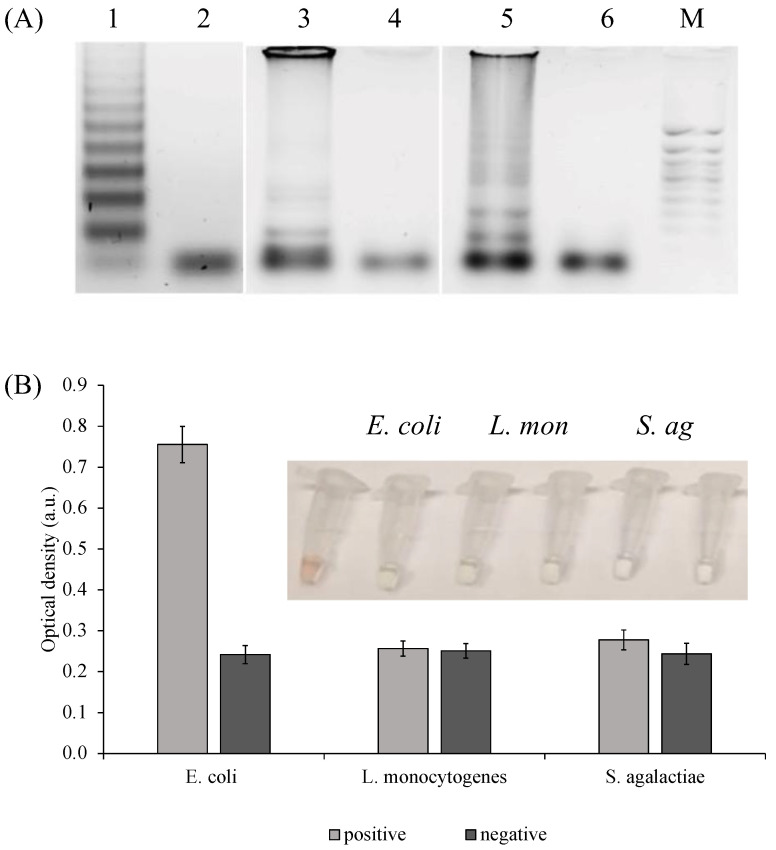
Specificity of *E. coli* PxD machine. (**A**) Amplification products analysis: 1—*E. coli*; 2—*E. coli* negative control; 3—*Listeria monocytogenes*; 4—*Listeria monocytogenes* negative control; 5—*Streptococcus agalactiae*; 6—*Streptococcus agalactiae* negative control; M—marker 50 bp+; (**B**) Colorimetric analysis of *E. coli*, *L. monocytogenes* (*L. mon*), and *S. agalactiae* (*S. ag*) amplicons (300 ng each) detection.

To evaluate the selectivity, DNA from various types of bacteria cells (*S. agalactiae, L. monocytogenes*, and *E. coli*), which are also pathogens of the central nervous system, were extracted, amplified, and analyzed (Figure 6A). *L. monocytogenes* and *S. agalactiae* shown were not detected by the sensor designed against *E. coli.(*Figure 6B).

**Figure 7:**
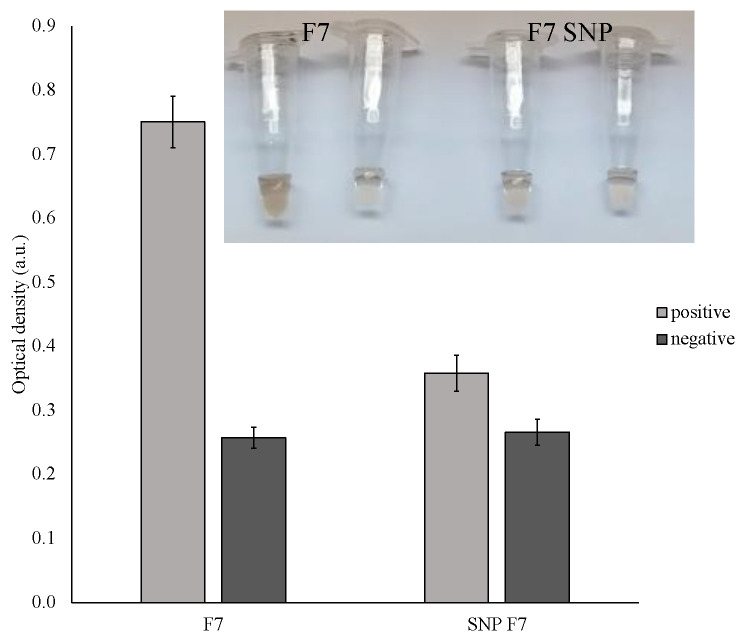
Single nucleotide selectivity of PxD machine. Specificity of F7 sensor against a single-base mismatched F7 sensor. A total of 300 ng of the amplicon was used for the detection. The mutation was presented in one of the sensing arms. Inset: the corresponding photograph of the color change.

To evaluate the visual biosensor ability to detect SNPs, a perfectly matched F7 and similar F7 sensor with a single-base mismatch was further investigated by comparing the absorbance response of the colorimetric system (Figure 7). The amount of staining was significantly lower when a mutated sensor was used. On the other hand, some coloration can be observed in the mismatched analyte and, therefore, if the sensor is supposed to be used in the mutation-prone region, the declared limit-of-detection should be carefully evaluated. More information and supporting experiments on single-base mismatch detection can be found in our previous studies [5,23].

### 2.6. PxDM Assay Does Not Detect False Positives

**Figure 8:**
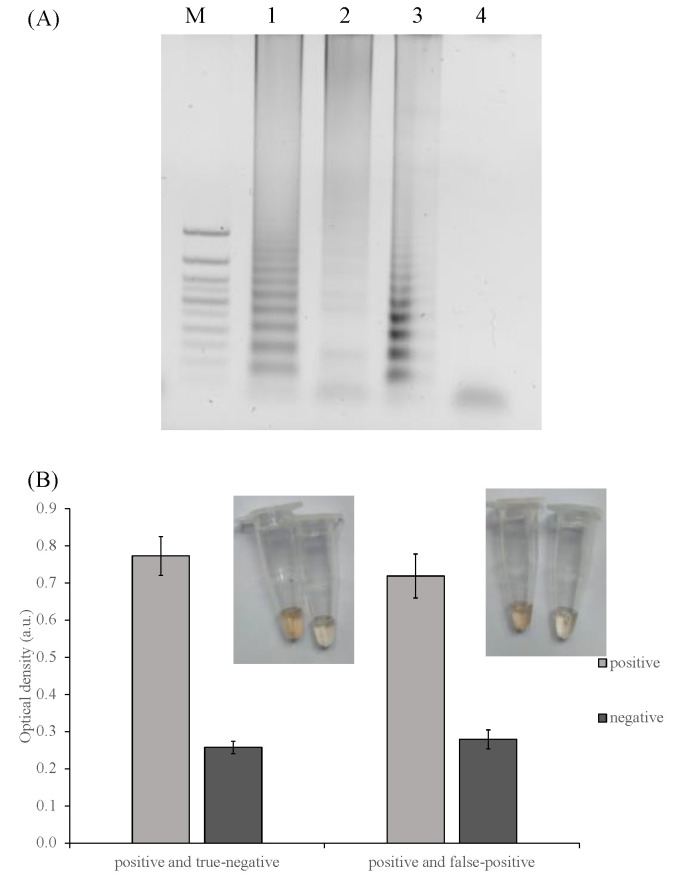
Discrimination of a true positive reaction. (**A**) SPA products analysis in 2% agarose: 1 and 3—*E. coli* true positive reaction: 2—false positive reaction; 4—true negative reaction; M—marker 50 bp+. (**B**) Colorimetric analysis of reaction buffer in the presence of true positive, false positive, and true negative *E. coli* amplicons.

To investigate if the PxDM can differentiate true from false positive samples, we prepared false positive samples by keeping negative SPA reactions at +20 °C overnight followed by the incubation of the samples under an amplification condition (65 °C, 1 h). Because the DNA polymerase used for SPA was not a hot start version, it starts its activity even at a lower temperature. Therefore, the prolonged incubation at a low temperature increases the chances of accumulation of the products of non-specific amplification. The electrophoretic analysis of the SPA reaction revealed amplification products in the negative (no DNA added) samples (Figure 8A, Lane 2). Still, PxDM was able to distinguish positive reactions from false positives with excellent accuracy (Figure 8).

### 2.7. Development of Diplex SPA and Detection of the SPA Products by PxDM Assay

One of current medical trends is to replace the singleplex with multiplex reactions. Such an approach reduces the cost and number of reactions to be completed for accurate diagnosis. The inventors of SPA [10] mentioned that the loop primers could have AA/TT or CC/GG dinucleotides at the end of the loops. To analyze the possibility of applying various dinucleotides for a diplex system, a set of Epstein–Barr virus primers was developed (see Table S1) and used for amplification in the singleplex format. Amplification products were analyzed in 2% agarose (Figure S1). The Epstein–Barr virus amplification products with primers with both dinucleotide sequences at the loop ends had identical expected amplification patterns and corresponding sizes on the gel.

The Epstein–Barr virus amplicons obtained with AA and CC primers were analyzed with PxDM designed for the EBV_DNA target (Table S2). The colorimetric analysis showed an identical optical density of both samples and S/B (Figure S1).

**Figure 9:**
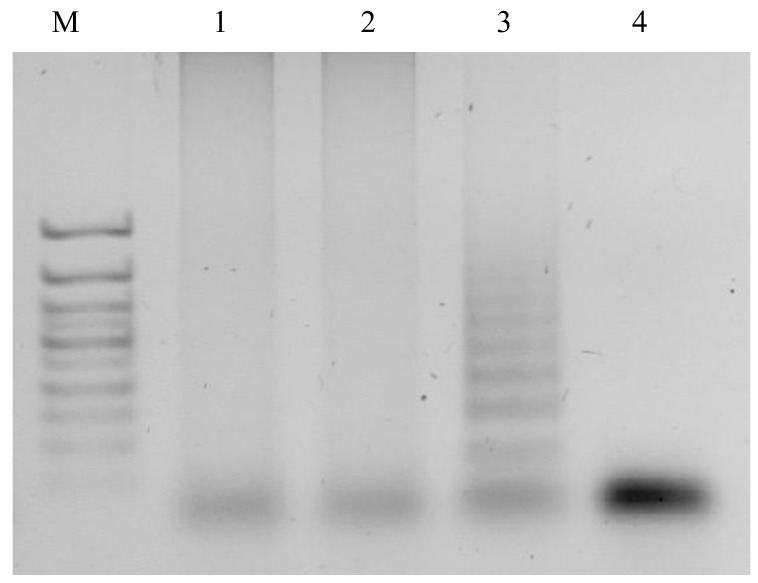
Diplex amplification of Epstein–Barr virus genome fragment and *E. coli* 16S rRNA: Lane M—marker 50 bp+; lane 1—AA E. coli/AA Epstein–Barr virus positive; lane 2—AA *E. coli*/AA Epstein–Barr virus negative control; lane 3—AA *E. coli*/CC Epstein–Barr virus positive; lane 4—AA *E. coli*/CC Epstein–Barr virus negative controls.

The SPA reaction was performed in a diplex format with primer sets AA *E. coli*/AA Epstein–Barr virus and AA *E. coli*/CC Epstein–Barr virus, and then the products of amplification were analyzed in 2% agarose (Figure 9). The results of the gel analysis showed amplicons of the expected patterns and sizes only for the duplex with different dinucleotides in the loop primers.

**Figure 10:**
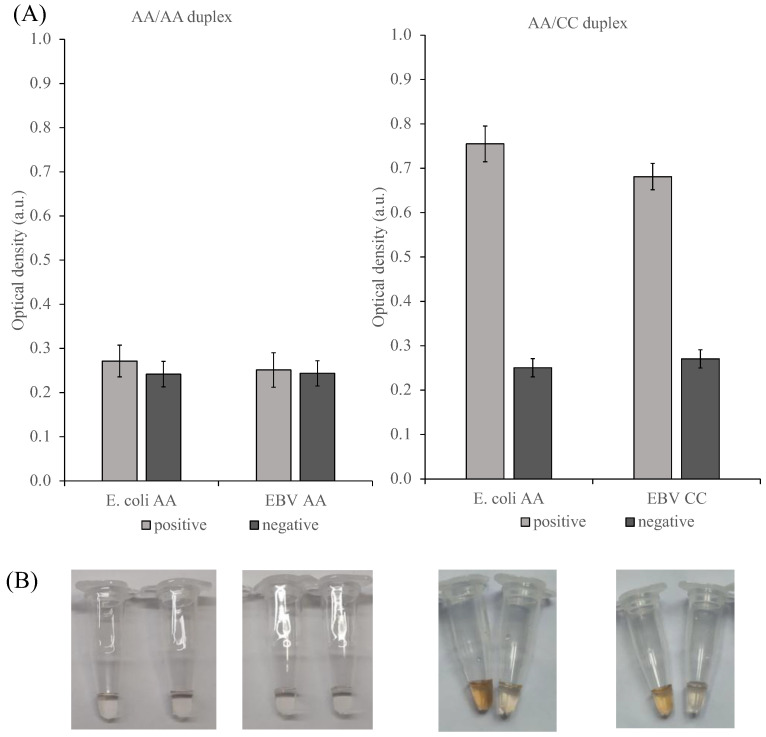
(**A**)AA *E. coli*/AA Epstein–Barr virus and AA *E. coli*/CC Epstein–Barr virus duplex amplicon analysis; (**B**) Corresponding photographs of the reaction tubes. The *E. coli* product was detected via the *E. coli* PxDM sensor and the EBV product was detected via the EBV PxDM sensor. Colorimetric analysis of reaction buffer in the presence (positive) or absence (negative) of 300 ng of purified SPA product. Strand concentrations: 1 μM of T1, 1 μM of T2, 1 μM of F7. Inset: the corresponding photograph of the color change.

The duplex amplification products with different pairs of dinucleotides in primers were analyzed using *E. coli* and the Epstein–Barr virus PxDM (Figure 10). The signals from negative controls and samples with amplification products of duplexes with identical dinucleotides in primers for *E. coli* and the Epstein–Barr virus were the same. However, it was shown that these pathogens can be distinguished in the case of the AA/CC primers combination, with the signal-to-background ratio ranging from 2.3 to 2.9.

Therefore, we have revealed the possibility of detecting *E. coli* and the Epstein–Barr virus in a duplex format.

## 3. Discussion

Double-stranded DNA amplicons are detected at high temperatures during a PCR cycle [24], after temperature denaturing [25] or after λ-exonuclease treatment [26]. We used DNA nanotechnology approaches to introduce the method that enables the detection of a dsDNA amplicon at ambient temperatures without the need for λ-exonuclease treatment.

The assay developed here is a combination of a stem-loop-primer-assisted isothermal amplification (SPA) and a multicomponent hybridization probe (PxDM). The PxDM forms the G-quadruplex structure after hybridization of the PxDM four arms to the target sequence followed by oxidation of a colorless substrate to a colored product visible by the naked eye.

Previously, we successfully applied this system to various samples, including the extracted RNA of the cell culture [27] or NASBA [5] or PCR amplicons [7]. All of these detectable products were single-stranded, and we technologically created the ability of PxDM to work with complex secondary structures only by flanking the core containing fragments with the additional unwinding arms. The know-how was initially tested on the deoxyribozyme sensor type [28] on chemically synthesized dsDNA, and this work presents a concept on such a complex product as SPA. We assume that even with the additional recognition fragments equipped, PxDMs are unable to work with relatively long dsDNA products. SPA can be coupled to PxDM due to its stringent limits of less than 150 bp in the fragment lengths. LAMP with PxDM cannot be employed as it is aimed at s working with longer fragments.

The PxDM-based visual detection of dsDNA sequences contributes to the development of a technology for point-of-care and home diagnostics. Technologies that use isothermal amplification and visual output signals may eliminate the need for equipment that requires thermal cycling and reading the fluorescent signals, which is likely to reduce diagnostic costs. At the same time, isothermal amplification proves to have excellent parameters of sensitivity and selectivity just like qPCR or digital PCR—the golden standards of diagnostics with the limit of detection reaching one genome equivalent [29].

The main drawback of isothermal amplification, and especially LAMP and SPA, is their susceptibility to false positive signals. The existing technologies such as turbidity [30] or ion detection [31] cannot visualize non-specific dsDNA amplicons, while the hybridization probes prevent miscomprehension and false positive results.

Hybridization probes coupled to qPCR or sequencing methods can detect SNPs; for example, they can be useful to assess antibiotic resistance in tuberculosis. These convenient methods require complex machinery, while the PxDMs on the amplicons can perform the SNP detection with a simple colorimetric visualization. This approach can be implemented to DNA chips or other heterogeneous user-friendly detection methods.

This work also presents the first multiplex version of the SPA technique. It is cost- effective as it minimizes manipulation having no denaturation step or λ exonuclease treatment, as well as amenable to multiplexing, as shown by the development of the duplex system in this study. An approach to creating a triplex and a more complex system is under development.

DNA nanomachines are currently at the early stages of their development, and despite the elevated levels of sensitivity in comparison to conventional beacon and Taqman probes, they still lack the limits of detection that are appropriate to regular clinical requirements and are provided by amplification techniques. On the other hand, the incorporation of the DNA nanomachines as a supportive tool to amplification techniques promise a perfect balance in the diagnostics preciseness and accessibility.

## 4. Materials and Methods

### 4.1. Materials

Amplification: dNTPs (10 mM of each, Evrogen, Moscow, Russia), RNase-free MQ water (Invitrogen, Waltham, MA, USA), Taq polymerase (Evrogen, Moscow, Russia), 10× Taq turbo buffer (Evrogen, Moscow, Russia), Bst DNA polymerase (NEB, Ipswich, MA, USA), 10× Thermopol Isothermal buffer (NEB, Ipswich, MA, USA), 100 mM magnesium sulfate (NEB, Ipswich, MA, USA), and betaine (Thermo Scientific, Waltham, MA, USA)

Electrophoresis: agarose LE 2 (Helicon, Moscow, Russia), ethidium bromide, 100+ bp DNA ladder, and 4× DNA loading buffer (Evrogen, Moscow, Russia).

Detection: Detection was performed by using the following reagents: COX buffer (50 mM HEPES, pH 7.4 (Helicon, Moscow, Russia), 50 mM MgCl_2_ (Helicon, Moscow, Russia), 70 mM KCl (Helicon, Moscow, Russia), 120 mM NaCl (Helicon, Moscow, Russia), 0.03% Triton X-100 (Helicon, Moscow, Russia), 1% DMSO (Helicon, Moscow, Russia)), 3,3′-Diaminobenzidine tetrahydrochloride (DAB), (Sigma-Aldrich, St. Loius, MI, USA), 30% hydrogen peroxide (Sigma-Aldrich, St. Loius, MI, USA), and hemin (Sigma-Aldrich, St. Loius, MI, USA) for detection reaction. A hemin stock solution was prepared in DMSO (Helicon, Moscow, Russia) and stored in the dark at −20 °C. DAB and H_2_O_2_ stock solutions were prepared in RNAse-free MQ water. All other chemicals were obtained from Helicon (Moscow, Russia).

All the oligonucleotides were purchased in Evrogen (Moscow, Russia) and DNA-synthesis (Moscow, Russia), by an individual order. Tables S1 and S2 depict the sequences of the oligonucleotides used in this study.

DNAse/RNAse-free water was purchased from QIAGEN, Germany and used for all stock solutions of oligonucleotides. MQ water was purified via Millipore RiOs-DI 3 Smart and used for buffers and solutions. The oligonucleotides were dissolved in DNAse/RNAse-free water and stored at −20 °C.

The data were processed using Microsoft Excel.

### 4.2. Cultivation of the Pathogens

The DNA of the Epstein–Barr virus was kindly presented by A.V. Slita from Pasteur’s Institute, Saint Petersburg, Russia. The Vero cell line (ECACC 84113001) was used to culture the Epstein–Barr virus. The line was maintained at 37 °C, 5% CO_2_, in a-MEM nutrient medium containing 10% fetal bovine serum (FBS, Thermo Scientific, Waltham, MA, USA ), penicillin, and streptomycin. Cells were infected in daily cultures when the monolayer thickness reached 80%.

*Streptococcus agalactiae*, *Listeria monocytogenes* were grown at 37 °C on Columbia agar with 5% sheep blood in 8–10% CO_2_ and obtained from the Institution Pediatric Research and Clinical Center for Infectious Diseases, Saint Petersburg, Russia. *E. coli* Nissle 1917 was cultured in LB (lysogeny broth) medium overnight at 37 °C in a shaker incubator Biosan (Riga, Latvia) with a rotation rate of 250 rpm. Media were sterilized by autoclaving at 121 °C for 40 min. Solid media were seeded by 100 μL of bacterial suspensions containing ~10^6^ cells that were spread over the surface of agar using a spatula. The number of samples was determined by plating serial 10-fold dilutions of the cell suspensions in 0.5% NaCl onto 1.5% Columbia agar after 1 day of incubation.

### 4.3. DNA Fragments Preparation

DNA was extracted using the phenol-chloroform method. A follow-up purification was performed with a Cleanup Standard kit (Evrogen, Moscow, Russia). The bacterial and cell suspensions were homogenized in 1 mL of the Extract RNA solution (Evrogen, Moscow, Russia) followed by RNA isolation according to the manufacturer’s instructions. Analysis of purified nucleic acids quantity and quality was performed by agarose gel electrophoresis and via spectrometry.

### 4.4. Primer and Sensor Design

Bacteria *Escherichia coli*, *Streptococcus agalactiae*, *Listeria monocytogenes* and the Epstein–Barr virus were selected as pathogens of the central nervous system. For the identification of bacteria, we have chosen specific genes that are constitutively expressed and selective for the particular organism; those were *E. coli*, a selective fragment of *16S rRNA*, the *S. agalactidae groEL* gene fragment, and part of the *Lmo0753* gene of *L.monocytogenes*. As for the viral agent, we have tested various genes related to the late lytic cycle mostly associated with proteins of a capsid and an envelope, and chose an analyte compatible with the gH gene’ fragment.

A set of LAMP primers was designed in PrimerExplorer v5 software [32], SPA, and PCR primers were designed using Unipro UGENE software 44.0 [33]. The primer set for the PCR consists of the forward primer (FP) and the backward primer (BP). The SPA primer mixture is proportionally composed of canonical PCR primers (Fr and Bv), and their stem-loop derivatives (stem-loop primers, FrSL, and BvSL). The primer set for LAMP consists of the forward inner primer (FIP), the backward inner primer (BIP), F3, and B3. All primers are shown in Table S1.

The scheme of the sensor is illustrated in Figure 1. The PxDM consists of stand F7 and two additional arms (T1 and T2) bound to the common DNA scaffold. Arms T1 and T2 are relatively long; they bind the analyte tightly and unwind its secondary structure. At the same time, the F7 strand remains detached from the dsDNA scaffold to ensure high binding selectivity. The system design is based on our earlier data [5] with the addition of the tile. The sensors were designed with the assistance of DinaMelt [34], mFold [35], and NuPack [36]. All sensors are presented in Table S2.

### 4.5. PCR Reaction

PCR was performed with a 25 μL reaction mixture containing 0.25 μM of each primer, 0.25 mM each dNTP, 2.5 μL of 10× Taq buffer, 5 U Taq polymerase, target DNA. The PCR conditions were as follows: 95 °C for 5 min, 30 cycles of denaturation (95 °C for 30 s), annealing (55 °C for 30 s), and elongation (72 °C for 10 s), followed by a final elongation at 72 °C for 10 min.

### 4.6. LAMP Reaction

LAMP was performed with a 25 μL reaction mixture containing 0.8 μM LAMP primer mixture (FIP:BIP:F3:B3 = 4:4:1:1 by concentration), 1.25 mM each dNTP, 0.8 M betaine, 2.5 μL of 10 × Isothermal amplification buffer, 8 mM MgSO4, 10 units of Bst DNA polymerase and target DNA. The LAMP reaction was generally performed at 63 °C for 90 min and terminated the reaction at 80 °C for 10 min. Before introducing the primer set to this work, they were tested to adjust the reaction parameters (temperature, Mg^2+^, and primer concentration) and the reaction specificity assessed via preforming the reaction on unspecific DNA.

### 4.7. SPA Reaction

SPA was performed in accordance with the authors’ recommendations [10], with 25 μL reaction mixture containing 0.8 μM SPA primer mixture (FrSL:BvSL:Fr:Bv = 4:4:1:1), 1.25 mM each dNTP, 0.8 M betaine, 2.5 μL of 10× Isothermal amplification buffer, 8 mM MgSO4, 10 units of Bst DNA polymerase and target DNA. The SPA reaction was generally performed at 63 °C for 90 min and terminated the reaction at 80 °C for 10 min. Before introducing the primer set to this work, they were tested to adjust the reaction parameters (temperature, Mg^2+^, and primer concentration) and the reaction specificity assessed via preforming the reaction on unspecific DNA.

### 4.8. Amplification Product Analysis

A nucleic acid analysis was performed by using 2% agarose gel electrophoresis at 80 V for 30 min. The ChemiDoc Imaging System (Bio-Rad, Hercules, CA, USA) was used for imaging and analyzing gels stained by ethidium bromide (Helicon, Moscow, Russia).

### 4.9. Optical Density Measurement/Colorimetric Detection/Visual Detection

The experiments were performed in 20 μL COX buffer (200 mM MgCl_2_, 150 mM KCl, 15 mM NaCl, and 50 mM HEPES pH 7.4), containing 1 μM of each PxDM strands (see Table S2 for sequences) and 1 μL of the amplicons. A total of 1 μM of hemin, 1 mM DAB, and 1 mM H_2_O_2_ were added, followed by a 20 min incubation at room temperature (23–25 °C).

In the case of the pre-denaturation experiment, the mixture of the PxDM strands with the amplicons was first incubated in 20 µL of COX buffer for 5 min at 95 °C followed by the addition of DAB, hemin, and H_2_O_2_, as described above.

A total of 1 μL of the mixture was collected, and the absorption spectra were obtained on a spectrophotometer N50 (Implen, Munich, Germany) at a wavelength of λ = 500 nm. All measurements were conducted at room temperature.

## 5. Conclusions

SPA isothermal amplification can detect various targets in combination with colorimetric PxDM without preheating the analyzed nucleic acids. PxDM combined with SPA demonstrates a sensitivity of up to 10 cells with the studied primer set, and a selectivity against other targets and to SNPs. Sensors provide protection to false positive results caused by an unspecific binding within the primer set. There is an option to create multiplex detection with changing the outer parts of the primer loops. This paper describes how combining SPA with G-quadruplex-based PxDM enables the development of a highly accurate and selective diagnostic approach. To the best of our knowledge, this is the first study that has demonstrated visual (color change) identification of SPA-produced dsDNA amplicons. Importantly, the established set of approaches allows the detection of isothermal DNA amplification products (LAMP, SPA), which extends the findings on the detection of the RNA isothermal amplification products (NASBA) [5]. The DNA amplicons are favored for detection as they are more stable than RNA under point-of-care settings.

The technology can be used for the visual detection of DNA fragments based on PxDM and isothermal amplification and allows diagnostics in the absence of specialized equipment and trained personnel. It also increases the signal/background ratio due to the absence of false positive accumulation of the amplification product associated with the nonspecific binding of primers. Further validation of this method with clinical samples and other analyte sequences is the next important step in assessing the practical value of the SPA/PxDM technology. With a thorough experiment design and with providing sufficient control testing and statistical implementation, the technology can be also used to detect single nucleotide polymorphisms without any fluorescent probing.

## Data Availability

No dataset created.

## Supplementary Materials

The supporting information can be downloaded at: https://www.mdpi.com/article/10.3390/ijms24097812/s1.
