# Supplementary material for: Visual Detection of Stem-Loop Primer Amplification (SPA) Products without Denaturation Using Peroxidase-like DNA Machines (PxDM)
Source: Int J Mol Sci. 2023 Apr 25;24(9):7812. doi: 10.3390/ijms24097812 (PMC10177805; doi:10.3390/ijms24097812)
Supplement: Supplementary file 1 [file ijms-24-07812-s001.zip › ijms-2325335-supplementary.pdf]

International Journal of
Molecular Sciences

MDPI

Supplementary Material

Peroxidase-like DNA machines (PxDM) for visual detection of stem-loop primer amplification (SPA) product

Yulia I. Maltzeva [1], Daria A. Gorbenko [1], Ekaterina V. Nikitina [2], Maria S. Rubel [1,*] and Dmitry M. Kolpashchikov [3,4,5]

[1] Laboratory of Solution Chemistry of Advanced Materials and Technologies, ITMO University, Lomonosova St. 9, 191002 St. Petersburg, Russian Federation; gorbenko@scamt-itmo.ru (D.A.G.); maltseva.yulia8@gmail.com (Y.I.M.); rubel@scamt-itmo.ru (M.S.R.)
[2] Pediatric Research and Clinical Center for Infectious Diseases, Prof. Popova St. 9, St Petersburg, Russian Federation; caterina.nikitina2010@yandex.ru
[3] Chemistry Department, University of Central Florida, 4000 Central Florida Boulevard, Orlando, FL 32816-2366, USA, dmitry.kolpashchikov@ucf.edu
[4] Burnett School of Biomedical Sciences, University of Central Florida, Orlando, 32816 Florida, USA
[5] National Center for Forensic Science, University of Central Florida, Orlando, FL, USA
* Correspondence: rubel@scamt-itmo.ru (M.S.R.)

Table of content:

1. **Oligonucleotides used in this study.**

2. **Analysis of EBV amplification products**

S1. Material and Methods

Table S1. Sequence of primers

Oligonucleotide name	Sequence 5’-3’
PCR E.coli primers	
F3	GCCATCTCCTGATGACGC
B3	ATTTACCGCAGCCAGACG
LAMP E.coli primers	
FIP	CTGGGGCGAGGTCGTCCTATTCCGACAAACACCACGAATT
BIP	CATTTTGCAGCTGTACGCTCGCAGCCCATCATGAATGTTGCT
F3	GCCATCTCCTGATGACGC
B3	ATTTACCGCAGCCAGACG
SPA *E. coli* primers	
FrSL_AA	TTTATATAATATATAAAAGGTTGTTACAAAGG
BvSL_AA	TTTATATAATATATAAACGACCTCGCCC
FrSL_CC	CCTATATAATATATAGGAGGTTGTTACAAAGG
BvSL_CC	CCTATATAATATATAGGCGACCTCGCCC
Fr	AGGTTGTTACAAAGG
Bv	CGACCTCGCC

SPA *St. agalactiae* primers	
Sagal_Fr	CCGGTAGAGCTATTACC
Sagal_Bv	AATGTGTTATCCCACTTC
Sagal_FrSL	TTTATATAATATATAAA CCGGTAGAGCTATTACC
Sagal_BvSL	TTTATATAATATATAAA AATGTGTTATCCCACTTC
SPA Epstein-Barr virus primers	
EBV_Frw	GTCTCGCCGGACTATGGCCT
EBV_Rw	TGTTCCAGCCACCCGCCATC
EBV_FrwSL	TTTATATAATATATAAA GTCTCGCCGGACTATGGCCT
EBV_RwSL	TTTATATAATATATAAA TGTTCCAGCCACCCGCCATC

Table S2. Sequence of the PxDMs

Oligonucleotide name	Sequence 5'-3'
Target E.coli DNA	ATAGTCAGCCCATCATGAATGTTGCTGTCGATGACAGGTTGTTACAAAGGGAGAAGGGCATGGCGAGCGTACAGCTGCAAAATGTAACGAAAGCCTGGGGCGAGGTCGTGGTATCGAAAGATATCAATCTCGATATCCATGAAGGTGAATTCGTGGTGTTTGTCGGACC
Target EBV DNA	TGGGCGGGCGTCTCGCCGGACTATGGCCTCGGCACGCTCGGCGTCGATGGCGGGTGGCTG GAACAGGCGGGCGAATGTGTAATCCCGGA
E.coli PxDM T1	CATATAAGGAATAGCAGAAGGCGGCTAG/HEG/CGTTACATTTTGCA/HEG/**GGGTAGGG**
E.coli PxDM T2	GACCTCGCCCCAGGCTTT/HEG/CTAGCCGCCTTCTGCTATTCCTTATATG/HEG/TGCCCTTCTCCCTTTGTAA
E.coli PxDM F7	**GGGTTGGG**/HEG/GCTGTACGCTCGCCAACC
F7 SNP	**GGGTTGGG**/HEG/GCTGTACACTCGCCAACC
EBV PxDM f7	**GGGTAGGG** tttt GCGTGCCGAGGCcc
EBV PxDM T1	ATTCGTAATGCTGACTAGATAGATtttccCGCCATCGACGCCGAtttt**GGGTTGGG**
EBV PxDM T2	TGTTCCAGCCACCtttttATCTATCTAGTCAGCATTACGAATtttttCATAGTCCGGCGAGAC

/HEG/- hexaethylene glycol linkers. Nucleotide linkers between different functional parts of PxDM are shown in low case. Bold nucleotides form G-quadruplex. Sequences of synthetic analytes corresponded to the sequences of amplicons. Red letter is a mismatched nucleotide introduced to the sensing arm of the sensor.

3. Analysis of EBV amplification products

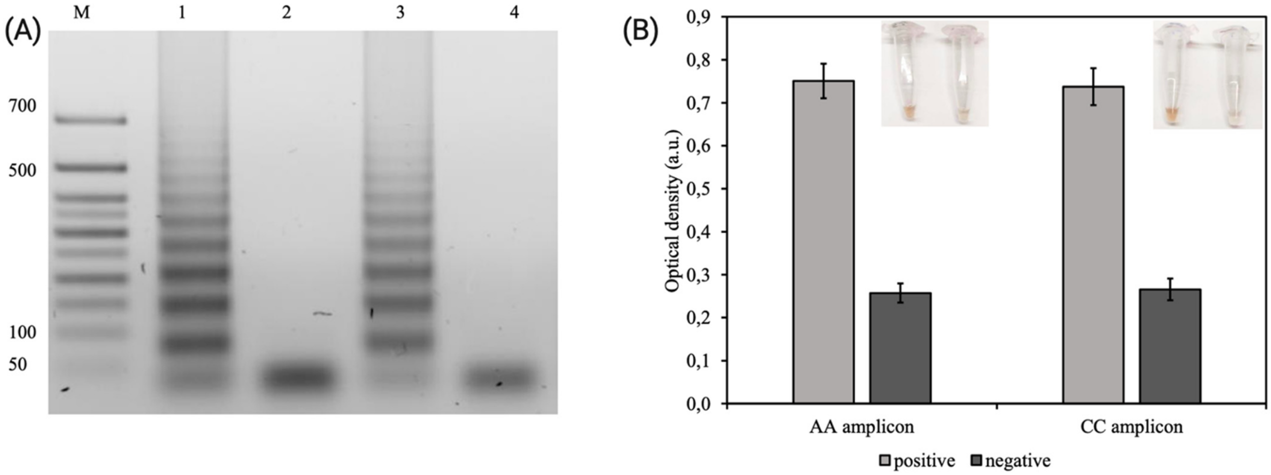

Figure S1. (A) An analysis of the EBV amplification products in 2% agarose. Analysis of Epstein-Barr virus amplicons obtained with AA and CC Epstein-Barr virus primers: M – marker 50 bp+, 1 – AA Epstein-Barr virus primers, 3 – CC Epstein-Barr virus primers, 2 and 4 – negative controls. (B) Analysis of Epstein-Barr virus amplicons obtained with AA and CC Epstein-Barr virus primers by Epstein-Barr virus PxDM. Colorimetric analysis of reaction buffer in the presence (positive) or absence (negative) of 300 ng of purified SPA product. Strand concentrations: 1 μM of T1, 1 μM of T2, 1 μM of F7. Inset: The corresponding photograph of the color change.
